# Transcriptome Analyses Implicate Endogenous Retroviruses Involved in the Host Antiviral Immune System through the Interferon Pathway

**DOI:** 10.1007/s12250-021-00370-2

**Published:** 2021-05-19

**Authors:** Miao Wang, Liying Wang, Haizhou Liu, Jianjun Chen, Di Liu

**Affiliations:** 1 CAS Key Laboratory of Special Pathogens and Biosafety, Wuhan Institute of Virology, Chinese Academy of Sciences, Wuhan, 430071 China; 2 Computational Virology Group, Center for Bacteria and Viruses Resources and Bioinformation, Wuhan Institute of Virology, Chinese Academy of Sciences, Wuhan, 430071 China; 3 University of Chinese Academy of Sciences, Beijing, 100049 China; 4 First Affiliated Hospital of Xinjiang Medical University, Urumqi, 830054 China

**Keywords:** Human endogenous retrovirus (HERV), RNA-seq, Virus infection, Interferon-stimulated genes (ISGs)

## Abstract

Human endogenous retroviruses (HERVs) are the remains of ancient retroviruses that invaded our ancestors’ germline cell and were integrated into the genome. The expression of HERVs has always been a cause for concern because of its association with various cancers and diseases. However, few previous studies have focused on specific activation of HERVs by viral infections. Our previous study has shown that dengue virus type 2 (DENV-2) infection induces the transcription of a large number of abnormal HERVs loci; therefore, the purpose of this study was to explore the relationship between exogenous viral infection and HERV activation further. In this study, we retrieved and reanalyzed published data on 21 transcriptomes of human cells infected with various viruses. We found that infection with different viruses could induce transcriptional activation of HERV loci. Through the comparative analysis of all viral datasets, we identified 43 key HERV loci that were up-regulated by DENV-2, influenza A virus, influenza B virus, Zika virus, measles virus, and West Nile virus infections. Furthermore, the neighboring genes of these HERVs were simultaneously up-regulated, and almost all such neighboring genes were interferon-stimulated genes (ISGs), which are enriched in the host’s antiviral immune response pathways. Our data supported the hypothesis that activation of HERVs, probably via an interferon-mediated mechanism, plays an important role in innate immunity against viral infections.

**Supplementary Information:**

The online version contains supplementary material available at 10.1007/s12250-021-00370-2.

## Introduction

Human endogenous retroviruses (HERVs) are derived from multiple independent integrations of exogenous retroviruses in our ancestor’s germ cell, most of which are more than 30 million years old (Bannert and Kurth 2006). After initial integration, some HERV elements were amplified within the genome via re-infection or intracellular retrotransposition (Dewannieux and Heidmann 2013); consequently, HERVs currently account for about 8% of the human genome (for protein-coding genes, about 1%) (Lander *et al.* 2001; Harrow *et al.* 2009). Over the course of co-evolution with the host, HERVs sequences have accumulated a large number of mutations, insertions, and deletions at the subject of natural selection (Gifford *et al.* 2018). Indeed, most HERVs in our genome contain no internal genes but solo-LTR (long-terminal repeat), which is a result of the recombination of 5′ and 3′ LTRs (Bolze *et al.* 2017). Therefore, the sequences of present-day HERVs are highly diverse and show defective replication. Although most HERVs are structurally incomplete and their activity is strictly controlled by epigenetic regulation (like DNA and histone methylation, histone deacetylation) (Hurst and Magiorkinis 2017; Fu *et al.* 2019) and 18-nt and 22-nt 3′-tRNA fragments (tRFs) (Schorn *et al.* 2017), transcriptions of some elements have been detected under a variety of physiological or pathological conditions. Traditional studies on HERVs have mostly focused on their relationship with cancer (Golan *et al.* 2008; Kahyo *et al.* 2013), pluripotency (Romer *et al.* 2017), and neurological diseases (Kury *et al.* 2018).

A few studies had found that there is a certain undefined correlation between HERVs and antiviral innate immunity. In animals, some endogenous retroviruses (ERVs) have been found to be co-opted by the host for resistance to the infection and replication of ERV-related exogenous viruses. For example, in mice, the *Fv-1* and *Fv-4* gene products derived from endogenous murine leukemia virus (MLV) provided resistance against exogenous MLV infection (Jolicoeur and Baltimore 1976; Ikeda and Odaka 1983; Taylor *et al.* 2001); in sheep, endogenous jaagsiekte sheep retrovirus (enJSRV) could suppress exogenous JSRV invasion (Spencer *et al.* 2003; Mura *et al.* 2004). Furthermore, it has been reported that transcription levels of *env* or *gag* genes of HERV-K or HERV-W increased significantly after infection by several viruses, like influenza A virus (Flu-A) (Nellaker *et al.* 2006), hepatitis B virus (HBV) (Liu *et al.* 2017), Epstein Barr virus (EBV) (Hsiao *et al.* 2009), and etc. Collectively, these studies suggest that some ERVs may have been integrated in the antiviral immune defense system of the host.

In addition, we previously reported the transcriptional profile of HERV loci in human A549 cells infected with dengue virus serotype 2 (DENV-2) (Wang *et al.* 2020); we found that a large number of HERV loci were up-regulated in DENV-2 infected cells. Interestingly, the reactivated loci tended to be distributed near immune-related genes and were expressed in a pattern consistent with that of the genes. On the basis of these findings, we defined a DEHERV-G pair as a pair of HERV and its nearest human gene (within 100 kb) on the same strand that was differentially expressed to describe the relationship between HERV reactivation and host genes under viral infection. However, it is necessary to explore whether the overall induction of the HERV loci is specific to DENV infection or a common phenomenon associated with viral infections. Although element activation of HERVs after viral infections had been detected using PCR amplification or microarray analysis in some studies (Muradrasoli *et al.* 2006; Tabone *et al.* 2018), the obtained results may not be comparable with those of other previous studies because the former targeted a limited number of members of the HERV family rather than the overall family. With the rapid development of RNA sequencing technology, RNA-seq datasets have become increasingly popular and are freely shared for scientific research; it is a convenient method for studying genome-wide transcriptional regulation of ERV under a range of physiological or pathological conditions.

In this study, we reanalyzed the published RNA-seq data corresponding to multiple viral infections that were downloaded from the NCBI database before March 31, 2020; we determined changes in the expression profiles of HERV induced by different viral infections. By comparing the related DEHERV-G pairs in each dataset, we identified 43 key HERV loci that were located near interferon-stimulated genes (ISGs) and were simultaneously up-regulated after exogenous viral infections. Our results provide evidence of the close association between HERVs and human antiviral responses.

## Materials and Methods

### RNA-Seq Datasets

We obtained RNA-Seq datasets of virus-infected human cells from the Sequence Read Archive (SRA) database (http://www.ncbi.nlm.nih.gov/sra/) using the SRA prefetch tool (v 2.9.3). The following accession numbers were used: PRJNA495858, PRJNA605905, PRJNA551246, PRJNA559203, PRJNA577579, PRJNA549926, PRJNA555053, PRJNA545099, PRJNA615032, and PRJNA609228. In addition, the transcriptomic findings of DENV-2 infected A549 cells reported in our previous study were included in the subsequent comparative analysis. Illumina RNA-seq reads, including those of single-end and double-end sequences, were used as expression data. Each dataset contained mock treated cells as the control, and each treatment was performed with 2–4 biological replicates. According to the conditions of viral infection, including the viral strain, time after infection, and cell type, abbreviations were assigned to the corresponding samples. For example, “HCV_12h_mock” represents the control sample for the sample that was analyzed 12 h after hepatitis C virus (HCV) infection. Summary of samples used in this study is shown in Table 1, and details of experiments and methods could be found in the original reports corresponding to the datasets.

**Table 1:** Summary of samples used in this study.

Virus	Hours post-infection	Virus strain	Cell	Accession number
DENV-2	72	TSV01	A549	PRJNA552644
Flu-A	–	PR8	A549	PRJNA495858
Flu-B	–	B/Yamagata	A549	PRJNA605905
ZIKV	48	FSS13025	hiNPCs	PRJNA551246
	48	PE243V	hiNPCs	
HCV	12	–	Huh7	PRJNA559203
	36	–	Huh7	
	60	–	Huh7	
WNV	–	–	A549	PRJNA577579
CCHFV	24	–	Huh7	PRJNA549926
	72	–	Huh7	
	24	–	HepG2	
	72	–	HepG2	
EBV	–	Akata	differentiated NOKs	PRJNA555053
	–	Akata	undifferentiated NOKs	
MV	–	–	MSC (5H)	PRJNA545099
	–	–	MSC (hTERT)	
RSV	24	A2	A549	PRJNA615032
SARS-CoV-2	24	USA-WA1/2020	A549	PRJNA615032
	24	USA-WA1/2020	NHBE	
RHV	6	–	Calu3	PRJNA609228
	24	–	Calu3	

### Reads Mapping and Counting

Data analyses were performed as described previously (Wang *et al.* 2020). Briefly, the quality of raw reads were assessed with FastQC (v 0.11.5) (Andrews 2010), and the low-quality reads were filtered out with Trimmomatic (v 0.38) (Bolger *et al.* 2014). The resulting reads were aligned upon the human genome GRCh38 with HISAT2 (v 2.1.0) (Kim *et al.* 2015), and counted using the "primary" option of featureCounts (v 1.6.3) (Liao *et al.* 2014), where only reads uniquely assigned to a single feature were counted. This approach may underestimate the total expression of features to some extent and slightly bias the expression values of older HERV elements, but allows convincing counts to be assigned to individual features. Conversely, if multiple mapped reads are not excluded, false positive expression of many undesired HERV loci may be detected. Therefore, using the unique mapped reads is more likely to avoid false positives for transcribed HERV loci. Ensembl GRCh38 version 95 GTF was used for annotated genes, while the GTF was derived from HERVd database containing the most extensive HERV loci for HERV loci annotation (Paces *et al.* 2004). In each experiment, features with less than 5 assigned reads across all samples were discarded.

### Differential Expression Analysis

The DESeq2 package was executed for differential expression analysis of human genes and HERV loci (Love *et al.* 2014). Adjusted *P* < 0.05 and | log2 fold change | > 1 were considered to be significant (Haase *et al.* 2015). When both HERV and its neighboring human gene were both differentially expressed (DE), they were collectively referred to as DEHERV-G pair. Subsequently, we calculated the number of DEHERV-G pairs in each dataset; we used *Venn* in TBtools to show their overlap and *Gene Locations* in TBtools to show the location of their components on chromosomes (Chen *et al.* 2020). The distribution of HERV loci relative to human genes (within exons, introns, UTRs, and 5 kb up- and down-stream regions) was determined using the *read distribution* module in the RSeQC package (v 3.0.1) (Wang *et al.* 2012).

### Construction of Protein–Protein Interaction (PPI) network

PPI network was constructed by using the Search Tool for the Retrieval of Interacting Genes/Proteins (STRING) database retrieval tool (http://stringdb.org/) (Szklarczyk *et al.* 2019). Interactors with a combined confidence score of ≥ 0.4 were selected, and based on these scores, the PPI network was illustrated using Cytoscape (Shannon *et al.* 2003).

### Functional Enrichment Analysis

The differentially expressed genes (DEGs) in the DEHERV-G pairs were subjected to Gene Ontology (GO) functional analysis and Kyoto Encyclopedia of Genes and Genomes (KEGG) pathway analysis using the clusterProfiler package (Yu *et al.* 2012). The GO categories include biological process (BP), cellular component (CC), and molecular function (MF). To reduce redundancy, the enrichGO analysis was followed by the “simplify” function as suggested by the author. Adjusted *P* < 0.05 was defined the threshold of statistical significance.

## Results

### RNA-Seq Datasets

As reported previously, we have observed that DENV infection induces HERVs activation. To explore whether this phenomenon is ubiquitous in viral infections, we used data obtained by the infection of human cells with multiple exogenous viruses to study the activation of HERV. Specifically, we performed a total of 179 SRA runs to download 21 high-throughput RNA-seq datasets from the SRA database and then re-analyzed them. These data covered 11 different viruses, including Flu-A, influenza B virus (Flu-B), Zika virus (ZIKV), HCV, measles virus (MV), West Nile virus (WNV), Crimean-Congo hemorrhagic fever virus (CCHFV), respiratory syncytial virus (RSV), rhinovirus (RHV), EBV, and severe acute respiratory syndrome coronavirus type 2 virus (SARS-CoV-2). In addition, we included our previously published DENV-2 infection data in this study for a comprehensive comparative analysis. The virus strains, infected cells, and time after infection of each sample are shown in Table 1.

After quality control of the raw reads, we obtained a total of about 1 TB of clean reads, and over 85% of reads, as a whole, were aligned with the human GRCh38 reference genome. Subsequently, using Ensembl GRCh38_95 GTF and the created HERV GTF as the annotation files, we detected human genes and HERVs expression in all analyzed samples. We found that approximately 1% of total reads corresponded to HERVs and that approximately 60% of total reads were mapped to human gene annotations. An overview of the RNA-Seq data is presented in Supplementary Table S1.

**Fig. 1:**
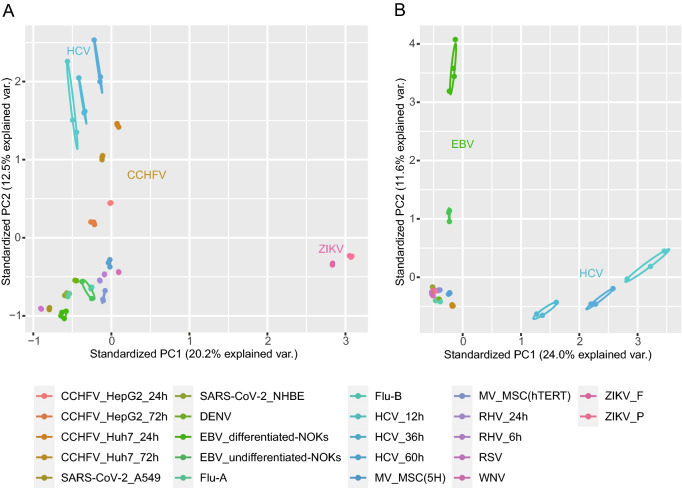
PCA analysis of the transformed expression values of human genes (**A**) and HERV loci (**B**).

The principal component analysis of the transformed expression of all 22 datasets is shown in Fig. 1. We found that samples were clustered within group and dispersed among the groups, and the samples of the same virus under different infection conditions were relatively closely distributed. For the expressions of human genes, HCV, CCHFV, and ZIKV groups were clearly subdivided from other virus samples. The overall expression of HERVs was similar in most datasets, whereas EBV and HCV samples were significantly different compared to the other groups.

### Identification of DE Human Genes and HERVs

Following conventional practices, we defined significantly differentially expressed human genes and HERV loci (DEGs and DEHERVs) as those with | log2 fold change | ≥ 1 with an adjusted *P* of ≤ 0.05. Data corresponding to DEGs and DEHERVs in all datasets are listed in Table 2. We found that the induction effects of some viruses, such as DENV-2 (4701 DEGs and 3033 DEHERVs), Flu-A (9130 DEGs and 6799 DEHERVs), and Flu-B (5544 DEGs and 2486 DEHERVs), were obvious. Compared to other respiratory viruses (like RSV with 659 DEGs and 75 DEHERVs), the SARS-CoV-2 USA-WA1/2020 strain (published in March 2020) had a weaker induction effect (33 DEGs and 6 DEHERVs in A549 cells, 127 DEGs, and 17 DEHERVs in normal human bronchial epithelial (NHBE) cells). A total of 30,549 DEGs and 16,707 DEHERVs were detected. After adjusting for redundancy, 16,248 DEGs and 11,427 DEHERVs were obtained, indicating that there was a crossover between the HERVs loci activated by different viral infections. In addition, correlation analysis showed that the number of DEHERVs was positively correlated with the number of DEGs (*R*^2^ = 0.9484) (Supplementary Fig. S1).

**Table 2:** Summary of differentially expressed human genes and HERVs in each dataset.

Dataset	Number of DEG^a^			Number of DEHERV^b^		
	Up-regulated	Down-regulated	Total	Up-regulated	Down-regulated	Total
DENV-2	2942	1759	4701	2352	681	3033
Flu-A	5341	3789	9130	4810	1989	6799
Flu-B	3667	1877	5544	1408	1078	2486
ZIKV_F	1072	274	1346	536	68	604
ZIKV_P	408	49	457	270	12	282
HCV_12h	4	4	8	0	0	0
HCV_36h	354	49	403	152	2	154
HCV_60h	1118	345	1463	208	33	241
WNV	825	52	877	349	6	355
CCHFV_Huh7_24h	12	0	12	6	0	6
CCHFV_Huh7_72h	559	348	907	147	61	208
CCHFV_HepG2_24h	44	1	45	20	0	20
CCHFV_HepG2_72h	121	77	198	45	8	53
EBV_differented-NOKs	237	772	1009	245	146	391
EBV_undifferented-NOKs	90	173	263	70	36	106
MV_MSC(5H)	1383	115	1498	1128	85	1213
MV_MSC(hTERT)	733	191	924	319	64	383
RSV	430	229	659	69	6	75
SARS-CoV-2_A549	32	1	33	6	0	6
SARS-CoV-2_NHBE	104	23	127	14	3	17
RHV_6h	1	0	1	2	1	3

^a^Differentially expressed human gene; ^b^differentially expressed HERV.

Subsequently, we compared the DEHERVs of each dataset in pairs. As shown in Supplementary Fig. S2A, the 22 datasets were clearly divided into two clusters, where EBV and HCV groups were significantly different from the other groups, which was similar to the expression of genome-wide HERV loci (Fig. 1B). Among them, ZIKV_F (FSS13025, isolated in Brazil in 2015) and ZIKV_P (PE243, a progenitor strain isolated in Cambodia in 2010) had the highest correlation coefficient, and 279 of 282 DEHERVs in the ZIKV_P group overlapped with those in the ZIKV_F group, indicating that the induction of HERVs by different strains was highly consistent. Moreover, the Flu-A and DENV groups shared the highest number of HERVs, followed by the Flu-A and Flu-B groups. In addition, the HCV, CCHFV, and RHV groups contained RNA-seq data obtained after different hours post infection (hpi); we found that exogenous viral infections affected the expressions of HERVs in a time-dependent manner. In the early stage of infection, the expressions of a small number of HERVs and host genes were changed, and the number of DEGs and DEHERVs was observed to increase with time (Supplementary Fig. S2B, Table 2). For example, relative to the mock treated group, 0, 154, and 241 DEHERVs in HCV-infected cells were detected at 12, 36, and 72 hpi, respectively.

It was worth noting that, with the exception of the major down-regulation of human genes in the two EBV groups, the number of up-regulated HERVs and genes was higher than the number of down-regulated HERVs and genes in cells infected with these viruses. Collectively, these results indicate that exogenous viral infections could not only stimulate host genes transcription, but also induce HERV activity in human cells.

### Identification of DEHERV-G Pairs in Each Dataset

**Fig. 2:**
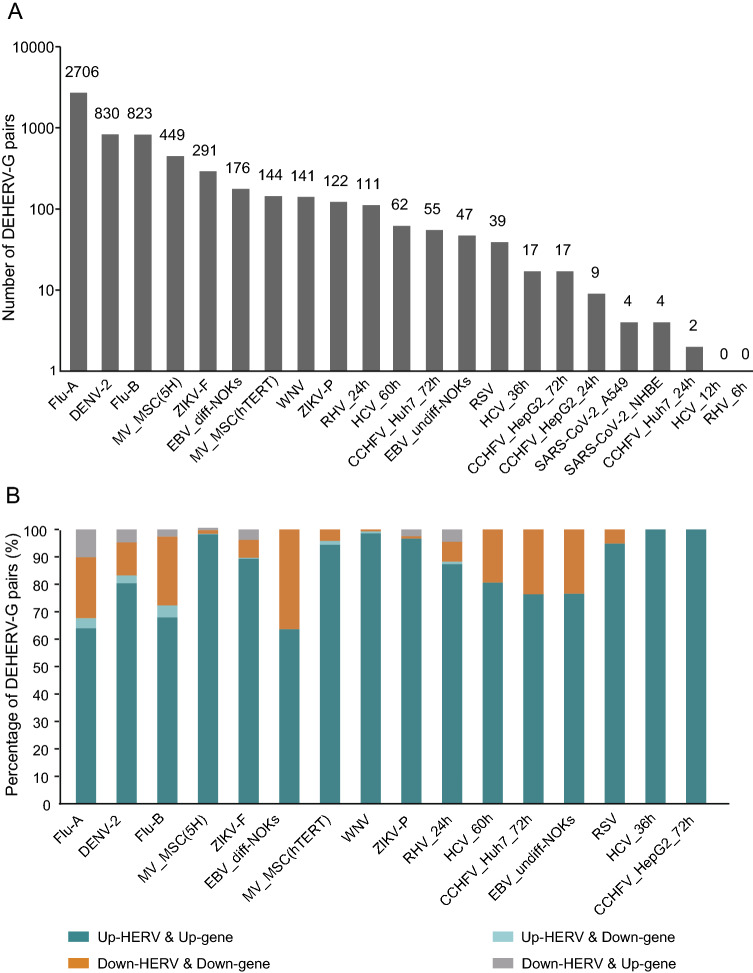
Identification of DEHERV-G pairs in each dataset. **A** Number of DEHERV-G pair. **B** Proportion of DEHERV-G pairs in four expression patterns. Only datasets with more than 10 DEHERV-G pairs are shown.

In our previous study, we found a correlation between viral infection-induced DEHERVs and DEGs and proposed the concept of DEHERV-G pair to reveal their potential interactions (Wang *et al.* 2020). In this study, we detected DEHERV-G pairs in each dataset. After adjusting for redundancy, only 4299 unique pairs were left out of the 6049 DEHERV-G pairs, indicating that the results of different datasets were partially overlapped. As shown in Fig. 2A, the datasets showing the highest number of DEHERV-G pairs were (in descending order) the Flu-A, DENV-2, Flu-B, MV_MSC(5H) (5H-mesenchymal stem cells), ZIKV_F, and EBV_diff-NOKs (differentiated normal oral keratinocytes (NOKs) cells) datasets, with 2706, 830, 823, 449, 291, and 176 pairs, respectively. Other datasets had relatively lower number of DEHERV-G pairs, for example, only four pairs were detected in both A549 and NHBE cells infected with SARS-CoV-2.

DEHERVs and DEGs, as components of DEHERV-G pairs, were divided into those showing up-regulated and down-regulated expression; therefore, there are four modes of DEHERV-G pair expression altogether. To avoid data bias, only the datasets with more than 10 pairs of DEHERV-Gs were subsequently analyzed (Fig. 2B). Interestingly, more than 90% of DEHERV-G pairs in all datasets had a consistent expression pattern, that is, both the expressions of their components were all up-regulated or down-regulated; up-regulation of HERVs and genes was mainly dominant.

### Screening of Key DEHERV-G Pairs

**Fig. 3:**
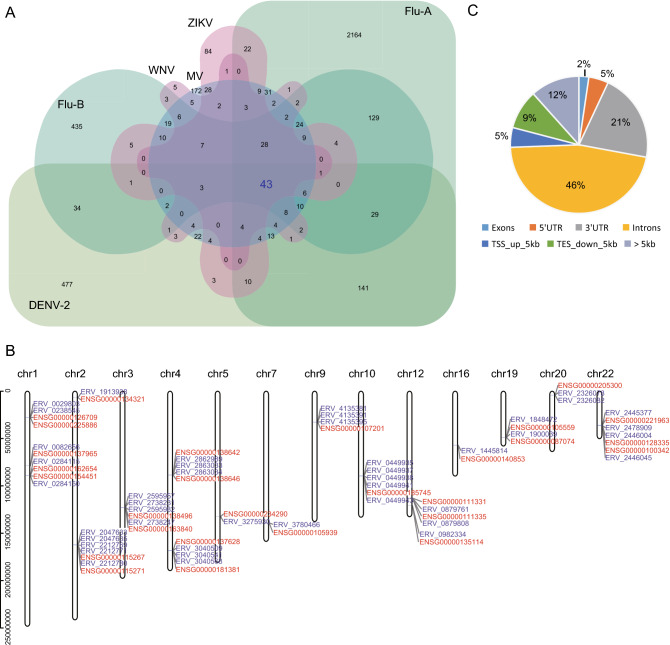
Screening for key DEHERV-G pairs associated with viral infection. **A** Veen diagram of key DEHERV-G pair enriched in six datasets. The pairs constituting the intersection of all datasets are considered to be the key DEHERV-G pairs.** B** Distribution of the components of the 43 key DEHERV-G pairs on chromosomes. The red letter represents human gene, and the blue letter represents HERV site. **C** Location of DEHERV loci relative to human genes. UTR: untranslated region; TSS: transcription start site; TES: transcription end site.

To explore the potential key HERV loci closely related to exogenous viral infections, we compared the DEHERV-G pairs detected in all datasets to screen for common DEHERV-G pairs in multiple datasets. Considering that the results of EBV datasets were very different from those of other datasets and that groups with a low number of elements would introduce bias into the intersection analysis, we analyzed DEHERV-G pairs that are common in DENV-2, Flu-A, Flu-B, ZIKV, WNV, and MV datasets (Fig. 3A). As listed in Supplementary Table S2, a total of 43 common DEHERV-G pairs were identified, which consisted of 43 HERVs and 28 human genes, and their structure is illustrated in Supplementary Fig. S3. Notably, all of their components were significantly up regulated. In term of distribution, these DEHERV-G pairs were not randomly distributed but were concentrated on certain autosomes, such as chr1, chr4, chr12, and chr22 (Fig. 3B). Furthermore, we analyzed the distance of HERV relative to its paired human gene (Fig. 3C). We found that almost all of the common 43 HERVs were located closely to their paired genes. About half of them were located in the intron region of neighboring gene, while only 2% of them were more than 5 kb away from the neighboring gene.

In addition, we analyzed the 43 common DEHERV-G pairs in all datasets (Supplementary Fig. S4). We found that, except for the EBV and HCV groups that did not contain these pairs, all other groups contained all or some of the 43 pairs. Moreover, the longer the time after infection, the greater the number of DEHERV-G pairs identified in the cells, which was consistent with previous results on the overall DEHERVs.

### Characteristics of the Components of Key DEHERV-G Pairs

**Fig. 4:**
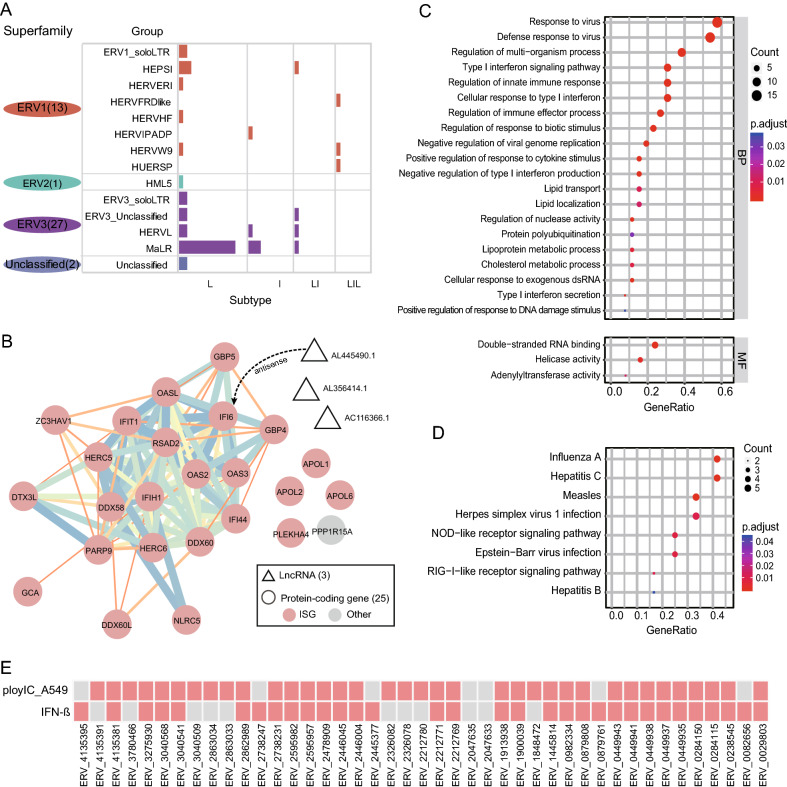
Characteristics of the components of 43 key DEHERV-G pairs. **A** Count of HERVs in different categories. The height of the columns represents the number of HERVs. **B** PPI network of human genes. The circle represents protein-coding gene, in which the red filled represents interferon induced genes, and the triangle represents lncRNA. **C** GO enrichment analysis of human genes. **D** KEGG enrichment analysis of human genes. **E** Distribution of the key DEHERV-G pairs in interform-beta or ploy(I:C)-stimulated cells.

Firstly, we analyzed the features of sequence type and classification of the 43 HERVs. As shown in Fig. 4A and Supplementary Table S2, 72% of the DEHERV loci were solo-LTRs (L: 31/43), 12% contained only internal sequences (I: 5/43), and the remaining loci contained single-terminal or double-terminal LTR sequences and internal gene sequences (LI: 4/43; LIL: 3/43). According to the method reported by Kojima (Kojima 2018), the common HERVs were mainly classified into the superfamily of ERV3 and ERV1, which accounted for about 63% (27/43) and 30% (13/43) of the HERVs, respectively. Among them, 18 loci belonged to the MaLR group of the ERV3 superfamily.

As another component, the human genes in the key DEHERV-G pairs included 3 long non-coding RNAs (lncRNAs) and 25 protein-coding genes (Fig. 4B, Supplementary Table S2). Literature review showed that 24 of the protein-coding genes were ISGs, and the expression of the remaining one gene, phosphatase 1 regulatory subunit 15A (*PPP1R15A*) gene, was reported to directly contribute to the stochasticity of interferon-beta (IFN-β) synthesis (Dalet *et al.* 2017). Additionally, expression of AL445490.1, which is called lncRNA-IFI6, was induced by IFN and could inhibit the expression of neighboring interferon alpha inducible protein 6 (IFI6) (Liu *et al.* 2019). STRING analysis showed that 20 of these genes were highly correlated with each other (Fig. 4B, Supplementary Table S3).

Finally, we performed functional and pathway enrichment analysis on the human genes of the 43 DEHERV-G pairs. GO enrichment analysis showed that they were mainly enriched in biological processes related to the innate immune response to viral infections, such as response to virus, type I interferon signaling pathway, and negative regulation of viral genome replication (adjusted *P* < 0.05) (Fig. 4C, Supplementary Table S4). In addition, KEGG pathway enrichment analysis showed that these genes were significantly enriched in a variety of signaling pathways related to viral infections, including Flu-A virus, HCV, MV, herpes simplex virus type 1, EBV, and hepatitis B virus (adjusted *P* < 0.05) (Fig. 4D, Supplementary Table S4). Furthermore, we taken corresponding analysis of transcriptomic data of IFN and ploy(I:C)-stimulated human-derived cells (accession numbers: PRJNA633674, PRJNA510831). Of all 43 key DEHERV-G pairs, 32 and 36 were found to be detected in IFN-β and ploy(I:C)-stimulated cells, respectively. Taken together, these results suggest that some HERVs are indeed closely associated with host immune response induced by viral infection and may have been integrated into the antiviral regulatory network underlying the IFN response.

## Discussion

HERVs account for a significant proportion of the human genome, far outnumbering all known protein-coding genes; therefore, their activation has considerable potential to influence physiological or pathological processes. Prior analysis of human cells by PCR or microarray methods had also indicated that transcription of different HERVs increases upon infection (Hsiao *et al.* 2009; Serafino *et al.* 2009; Liu *et al.* 2017). Considering the poor conservation and complex structure of HERVs, traditional methods cannot elucidate the expression profile of HERV loci in the whole genome, which is not conducive to the comparison of the similarities and differences between HERVs in different studies and to the exploration of the role of their activation in antiviral response. However, previous high-throughput sequencing studies on physiological innate immune responses of the host predominantly highlighted the responsiveness of gene transcription but lacked the widespread expression of HERVs. Therefore, in this study, we decided to fully use the published RNA-seq data on virus-infected cells to assess the modulation of species HERV transcription in innate immune.

In a previous study, we reported the transcription profile of HERVs in DENV-2 infected A549 cells using high-throughput sequencing for the first time and found the correlation between activated HERV sites and human genes (Wang *et al.* 2020). In this study, using the same pipeline, we reanalyzed the RNA-sequencing data associated with 11 viral infections, including those of Flu-A, Flu-B, ZIKV, HCV, CCHFV, RSV, SARS-CoV-2, WNV, RHV, MV, and EBV. We found that most expressed HERVs appeared to be up regulated in human cells upon infection, which is in line with that of human genes. Moreover, our PCA analysis showed that the expressed HERVs of EBV groups were far away from those of other viruses, suggesting that HERVs driven by DNA or RNA viral infection may be different. However, owing to the relative dearth of published RNA-seq data on DNA virus infection in human cells, follow-up experiments are needed to reveal the differences in HERV activation between DNA virus and RNA virus infection further.

Importantly, we observed overlaps between HERV activation induced by multiple viral infections and finally identified 43 key HERVs. Most of them were members of the older superfamily of ERV1 and ERV3, while only one HERV belonged to the younger ERV2 superfamily. Interestingly, these HERVs were mainly distributed near ISGs, and they were significantly up-regulated in all six different virus-infected cells. In fact, it has been reported that the MER41 elements, a subset of mouse ERVs, contributed to IRF1 (interferon regulatory factor 1) and STAT (signal transducer and activator of transcription 1) binding sites to affect the expression of adjacent IFN-induced genes, such as absent in melanoma 2 (AIM2), apolipoprotein L1 (APOL1), IFI6, and secreted and transmembrane 1 (SECTM1) (Chuong *et al.* 2016). Consistent with the report, we also detected that ERV_2446004 and ERV_2446045 were neighbored by APOL1, while ERV_0238545 was neighbored by IFI6; these ERVs were among the 43 pairs that were closely related to viral infections. Additionally, we identified three lncRNAs in the common DEHERV-G pairs: AL356414.1, AL445490.1, and AC116366.1. Indeed, AL445490.1, which is the antisense sequence of IFI6, has been described to regulate the expression of neighboring genes to affect HCV replication (Liu *et al.* 2019; Unfried and Fortes 2020). In addition, AL356414.1 was reported to be a prognosis-related lncRNA of laryngeal squamous cell carcinoma (Cui *et al.* 2019), while AC116366.1 could reflect the immune infiltrating status of breast cancer (Li *et al.* 2020), and they were found to be associated with viral infection in this study. Collectively, these results suggest that some particular old HERVs may play a role in the induction of innate immune response as well as in the evolution of host interferon regulatory network. We predicted that activated HERVs may act as enhancer elements to directly affect the expression of adjacent ISGs and may regulate the expression of nearby lncRNAs to affect the antiviral immune response network, although the mechanisms require further exploration.

Moreover, we observed differences in the activation of HERVs under different infection conditions, such as time after infection and cell type, as in the case of modulation of host genes (Eichelberg *et al.* 2019). Among them, the viral strains (e.g., two ZIKV strains) affected the induction of HERVs activation. Consistent with this finding, the inflammatory response against ZIKV_P was weaker than that against ZIKV_F (Lima *et al.* 2019); we found the number of DEHERVs that were activated by ZIKV_F infection was small, and almost all of them were included in the results of FSS13025 strain, with lower expression levels than the latter. In addition, based on the different distribution of key HERV-G pairs, i.e., 35 pairs in the ZKV-P group and 43 pairs in the ZIKV_F group, we speculated that the difference in the activation of HERVs induced by different strains may contribute to the difference in immune response. Further research is needed to verify this hypothesis. Considering that the datasets used in this study were collected from different experiments and that different operations may introduce some deviations, some of our findings require further molecular experimental verification, such as maintenance of uniform infection conditions to compare the impact of multiple viral infections.

In summary, we reanalyzed the expression profiles of HERVs and human genes in 11 virus-infected RNA-seq datasets. We found that exogenous viral infections can generally induce HERV expression, while the transcriptional activation of HERV showed similarities and differences between different datasets. In addition, we found 43 HERVs that were commonly expressed by several viral infections were closely related to ISGs. Further studies are needed to determine the molecular mechanism by which these HERVs are involved in the elicitation of innate immune response.

## Supplementary Information

Below is the link to the electronic supplementary material.

Supplementary file1 (PDF 297 kb)

Supplementary file2 (ZIP 59 kb)

## References

[CR1] Andrews S (2010) FastQC: a quality control tool for high throughput sequence data.

[CR2] Bannert N, Kurth R (2006). The evolutionary dynamics of human endogenous retroviral families. Annu Rev Genomics Hum Genet.

[CR3] Bolger AM, Lohse M, Usadel B (2014). Trimmomatic: a flexible trimmer for Illumina sequence data. Bioinformatics.

[CR4] Bolze PA, Mommert M, Mallet F (2017). Contribution of syncytins and other endogenous retroviral envelopes to human placenta pathologies. Prog Mol Biol Transl Sci.

[CR5] Chen C, Chen H, Zhang Y, Thomas HR, Frank MH, He Y, Xia R (2020). TBtools: an integrative toolkit developed for interactive analyses of big biological data. Mol Plant.

[CR6] Chuong EB, Elde NC, Feschotte C (2016). Regulatory evolution of innate immunity through co-option of endogenous retroviruses. Science.

[CR7] Cui J, Wen Q, Tan X, Chen Z, Liu G (2019). A genomic-clinicopathologic nomogram predicts survival for patients with laryngeal squamous cell carcinoma. Dis Markers.

[CR8] Dalet A, Arguello RJ, Combes A, Spinelli L, Jaeger S, Fallet M, Vu Manh TP, Mendes A, Perego J, Reverendo M, Camosseto V, Dalod M, Weil T, Santos MA, Gatti E, Pierre P (2017). Protein synthesis inhibition and GADD34 control IFN-beta heterogeneous expression in response to dsRNA. EMBO J.

[CR9] Dewannieux M, Heidmann T (2013). Endogenous retroviruses: acquisition, amplification and taming of genome invaders. Curr Opin Virol.

[CR10] Eichelberg MR, Welch R, Guidry JT, Ali A, Ohashi M, Makielski KR, McChesney K, Van Sciver N, Lambert PF, Keles S, Kenney SC, Scott RS, Johannsen E (2019) Epstein-Barr Virus infection promotes epithelial cell growth by attenuating differentiation-dependent exit from the cell cycle. mBio 10:e01332–01319 10.1128/mBio.01332-19 PMC6703421 31431547

[CR11] Fu B, Ma H, Liu D (2019). Endogenous retroviruses function as gene expression regulatory elements during mammalian pre-implantation embryo development. Int J Mol Sci.

[CR12] Gifford RJ, Blomberg J, Coffin JM, Fan H, Heidmann T, Mayer J, Stoye J, Tristem M, Johnson WE (2018). Nomenclature for endogenous retrovirus (ERV) loci. Retrovirology.

[CR13] Golan M, Hizi A, Resau JH, Yaal-Hahoshen N, Reichman H, Keydar I, Tsarfaty I (2008). Human endogenous retrovirus (HERV-K) reverse transcriptase as a breast cancer prognostic marker. Neoplasia.

[CR14] Haase K, Moesch A, Frishman D (2015). Differential expression analysis of human endogenous retroviruses based on ENCODE RNA-seq data. BMC Med Genomics.

[CR15] Harrow J, Nagy A, Reymond A, Alioto T, Patthy L, Antonarakis SE, Guigo R (2009). Identifying protein-coding genes in genomic sequences. Genome Biol.

[CR16] Hsiao FC, Tai AK, Deglon A, Sutkowski N, Longnecker R, Huber BT (2009). EBV LMP-2A employs a novel mechanism to transactivate the HERV-K18 superantigen through its ITAM. Virology.

[CR17] Hurst TP, Magiorkinis G (2017). Epigenetic Control of Human Endogenous Retrovirus Expression: Focus on Regulation of Long-Terminal Repeats (LTRs). Viruses.

[CR18] Ikeda H, Odaka T (1983). Cellular expression of murine leukemia virus gp70-related antigen on thymocytes of uninfected mice correlates with Fv-4 gene-controlled resistance to friend leukemia virus infection. Virology.

[CR19] Jolicoeur P, Baltimore D (1976). Effect of Fv-1 gene product on proviral DNA formation and integration in cells infected with murine leukemia viruses. Proc Natl Acad Sci USA.

[CR20] Kahyo T, Tao H, Shinmura K, Yamada H, Mori H, Funai K, Kurabe N, Suzuki M, Tanahashi M, Niwa H, Ogawa H, Tanioka F, Yin G, Morita M, Matsuo K, Kono S, Sugimura H (2013). Identification and association study with lung cancer for novel insertion polymorphisms of human endogenous retrovirus. Carcinogenesis.

[CR21] Kim D, Langmead B, Salzberg SL (2015). HISAT: a fast spliced aligner with low memory requirements. Nat Methods.

[CR22] Kojima KK (2018) Human transposable elements in Repbase: genomic footprints from fish to humans. Mobile DNA 9 10.1186/s13100-017-0107-y PMC5753468 29308093

[CR23] Kury P, Nath A, Creange A, Dolei A, Marche P, Gold J, Giovannoni G, Hartung HP, Perron H (2018). Human endogenous retroviruses in neurological diseases. Trends Mol Med.

[CR24] Lander ES, Sequencing IHG, C, Linton LM, Birren B, Nusbaum C, Zody MC, Baldwin J, Devon K, Dewar K, Doyle M *et al*. (2001) Initial sequencing and analysis of the human genome. Nature 409:860–921 10.1038/35057062 11237011

[CR25] Li Z, Li Y, Wang X, Yang Q (2020). Identification of a six-immune-related long non-coding RNA signature for predicting survival and immune infiltrating status in breast cancer. Front Genet.

[CR26] Liao Y, Smyth GK, Shi W (2014). featureCounts: an efficient general purpose program for assigning sequence reads to genomic features. Bioinformatics.

[CR27] Lima MC, de Mendonca LR, Rezende AM, Carrera RM, Anibal-Silva CE, Demers M, D'Aiuto L, Wood J, Chowdari KV, Griffiths M, Lucena-Araujo AR, Barral-Netto M, Azevedo EAN, Alves RW, Farias PCS, Marques ETA, Castanha PMS, Donald CL, Kohl A, Nimgaonkar VL, Franca RFO (2019). The transcriptional and protein profile from human infected neuroprogenitor cells is strongly correlated to Zika Virus Microcephaly Cytokines phenotype evidencing a persistent inflammation in the CNS. Front Immunol.

[CR28] Liu C, Liu L, Wang X, Liu Y, Wang M, Zhu F (2017). HBV X Protein induces overexpression of HERV-W env through NF-kappa B in HepG2 cells. Virus Genes.

[CR29] Liu X, Duan X, Holmes JA, Li W, Lee SH, Tu Z, Zhu C, Salloum S, Lidofsky A, Schaefer EA, Cai D, Li S, Wang H, Huang Y, Zhao Y, Yu ML, Xu Z, Chen L, Hong J, Lin W, Chung RT (2019). A long noncoding RNA regulates Hepatitis C Virus infection through interferon alpha-inducible Protein 6. Hepatology.

[CR30] Love MI, Huber W, Anders S (2014). Moderated estimation of fold change and dispersion for RNA-seq data with DESeq2. Genome Biol.

[CR31] Mura M, Murcia P, Caporale M, Spencer TE, Nagashima K, Rein A, Palmarini M (2004). Late viral interference induced by transdominant Gag of an endogenous retrovirus. Proc Natl Acad Sci USA.

[CR32] Muradrasoli S, Forsman A, Hu L, Blikstad V, Blomberg J (2006). Development of real-time PCRs for detection and quantitation of human MMTV-like (HML) sequences HML expression in human tissues. J Virol Methods.

[CR33] Nellaker C, Yao Y, Jones-Brando L, Mallet F, Yolken RH, Karlsson H (2006). Transactivation of elements in the human endogenous retrovirus W family by viral infection. Retrovirology.

[CR34] Paces J, Pavlicek A, Zika R, Kapitonov VV, Jurka J, Paces V (2004). HERVd: the Human Endogenous RetroViruses database: update. Nucleic Acids Res.

[CR35] Romer C, Singh M, Hurst LD, Izsvak Z (2017). How to tame an endogenous retrovirus: HERVH and the evolution of human pluripotency. Curr Opin Virol.

[CR36] Schorn AJ, Gutbrod MJ, LeBlanc C, Martienssen R (2017). LTR-Retrotransposon control by tRNA-derived small RNAs. Cell.

[CR37] Serafino A, Balestrieri E, Pierimarchi P, Matteucci C, Moroni G, Oricchio E, Rasi G, Mastino A, Spadafora C, Garaci E, Vallebona PS (2009). The activation of human endogenous retrovirus K (HERV-K) is implicated in melanoma cell malignant transformation. Exp Cell Res.

[CR38] Shannon P, Markiel A, Ozier O, Baliga NS, Wang JT, Ramage D, Amin N, Schwikowski B, Ideker T (2003). Cytoscape: a software environment for integrated models of biomolecular interaction networks. Genome Res.

[CR39] Spencer TE, Mura M, Gray CA, Griebel PJ, Palmarini M (2003). Receptor usage and fetal expression of ovine endogenous betaretroviruses: Implications for coevolution of endogenous and exogenous retroviruses. J Virol.

[CR40] Szklarczyk D, Gable AL, Lyon D, Junge A, Wyder S, Huerta-Cepas J, Simonovic M, Doncheva NT, Morris JH, Bork P, Jensen LJ, Mering C (2019). STRING v11: protein-protein association networks with increased coverage, supporting functional discovery in genome-wide experimental datasets. Nucleic Acids Res.

[CR41] Tabone O, Mommert M, Jourdan C, Cerrato E, Legrand M, Lepape A, Allaouchiche B, Rimmele T, Pachot A, Monneret G, Venet F, Mallet F, Textoris J (2018). Endogenous retroviruses transcriptional modulation after severe infection. Trauma Burn Front Immunol.

[CR42] Taylor GM, Gao Y, Sanders DA (2001). Fv-4: Identification of the defect in Env and the mechanism of resistance to ecotropic murine leukemia virus. J Virol.

[CR43] Unfried JP, Fortes P (2020) LncRNAs in HCV Infection and HCV-Related Liver Disease. Int J Mol Sci 21 10.3390/ijms21062255 PMC7139329 32214045

[CR44] Wang L, Wang S, Li W (2012). RSeQC: quality control of RNA-seq experiments. Bioinformatics.

[CR45] Wang M, Qiu Y, Liu H, Liang B, Fan B, Zhou X, Liu D (2020). Transcription profile of human endogenous retroviruses in response to dengue virus serotype 2 infection. Virology.

[CR46] Yu G, Wang L-G, Han Y, He Q-Y (2012). clusterProfiler: an R package for comparing biological themes among gene clusters. Omics J Integrative Biol.

