# Supplementary material for: Transcriptome Analyses Implicate Endogenous Retroviruses Involved in the Host Antiviral Immune System through the Interferon Pathway
Source: Virol Sin. 2021 May 19;36(6):1315–26. doi: 10.1007/s12250-021-00370-2 (PMC8131884; doi:10.1007/s12250-021-00370-2)
Supplement: Supplementary file 1 — Supplementary file1 (PDF 297 kb) [file 12250_2021_370_MOESM1_ESM.pdf]

VIROLOGICA SINICA

Electronic Supplementary Material

Transcriptome Analyses Implicate Endogenous Retroviruses Involved in the Host Antiviral Immune System through the Interferon Pathway

Miao Wang[1,2,3] • Liying Wang[1,2,3] • Haizhou Liu[1,2] • Jianjun Chen[1,2] • Di Liu[1,2,3,4]✉

1. CAS Key Laboratory of Special Pathogens and Biosafety, Wuhan Institute of Virology, Chinese Academy of Sciences, Wuhan 430071, China
2. Computational Virology Group, Center for Bacteria and Viruses Resources and Bioinformation, Wuhan Institute of Virology, Chinese Academy of Sciences, Wuhan 430071, China
3. University of Chinese Academy of Sciences, Beijing 100049, China
4. First Affiliated Hospital of Xinjiang Medical University, Urumqi 830054, China

Supporting information to DOI: 10.1007/s12250-021-00370-2

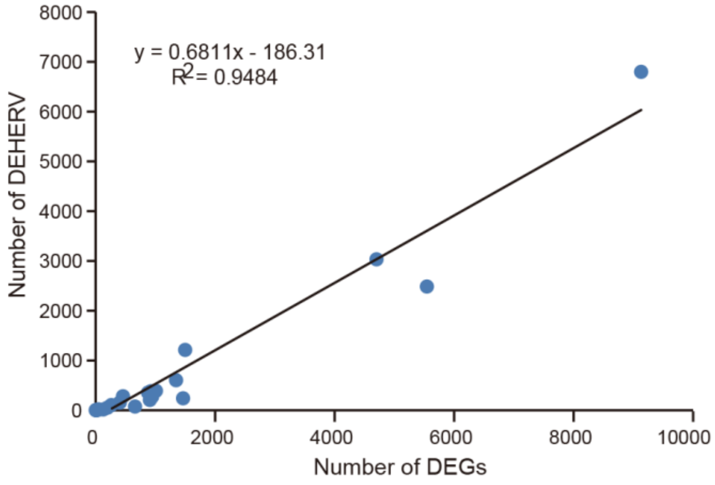

Fig. S1 Correlation between the number of differentially expressed genes and that of HERVs.

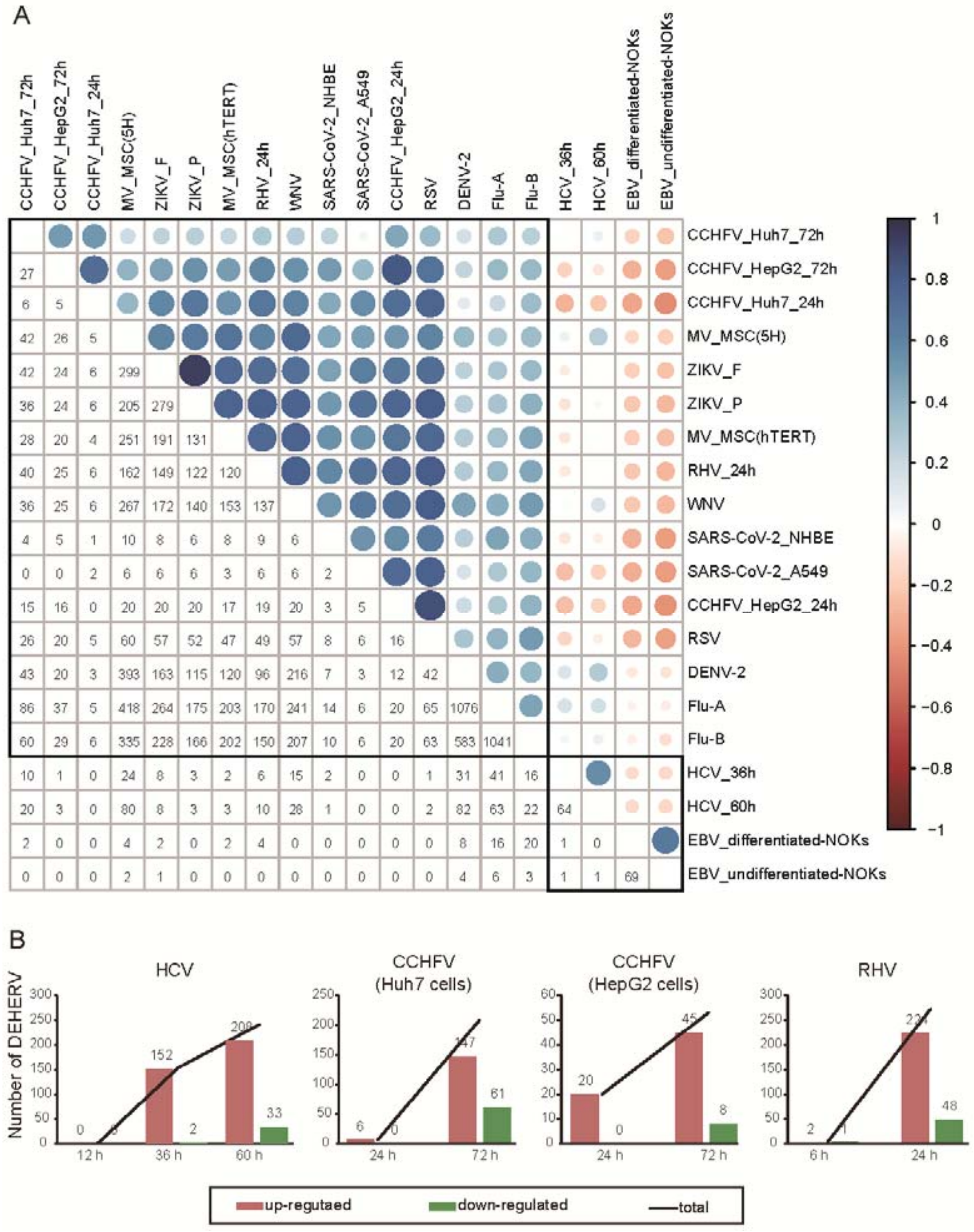

Fig. S2 Correlation between differentially expressed HERVs in each dataset. **A** Correlation matrix between the DEHERVs of each dataset. Correlation values are presented in the upper panel in circle, where color intensity (light to dark) and the size of the circle (small to big) are proportional to the correlation coefficients. The numbers of overlapping DEHERVs of datasets in pairs are listed in the lower panel. **B** Number of DEHERV loci after different post-infection periods for HCV, CCHFV, and RHV. The red and green bars represent the number of up-regulated and down-regulated genes or HERV, respectively, and the gray lines represent the total numbers.

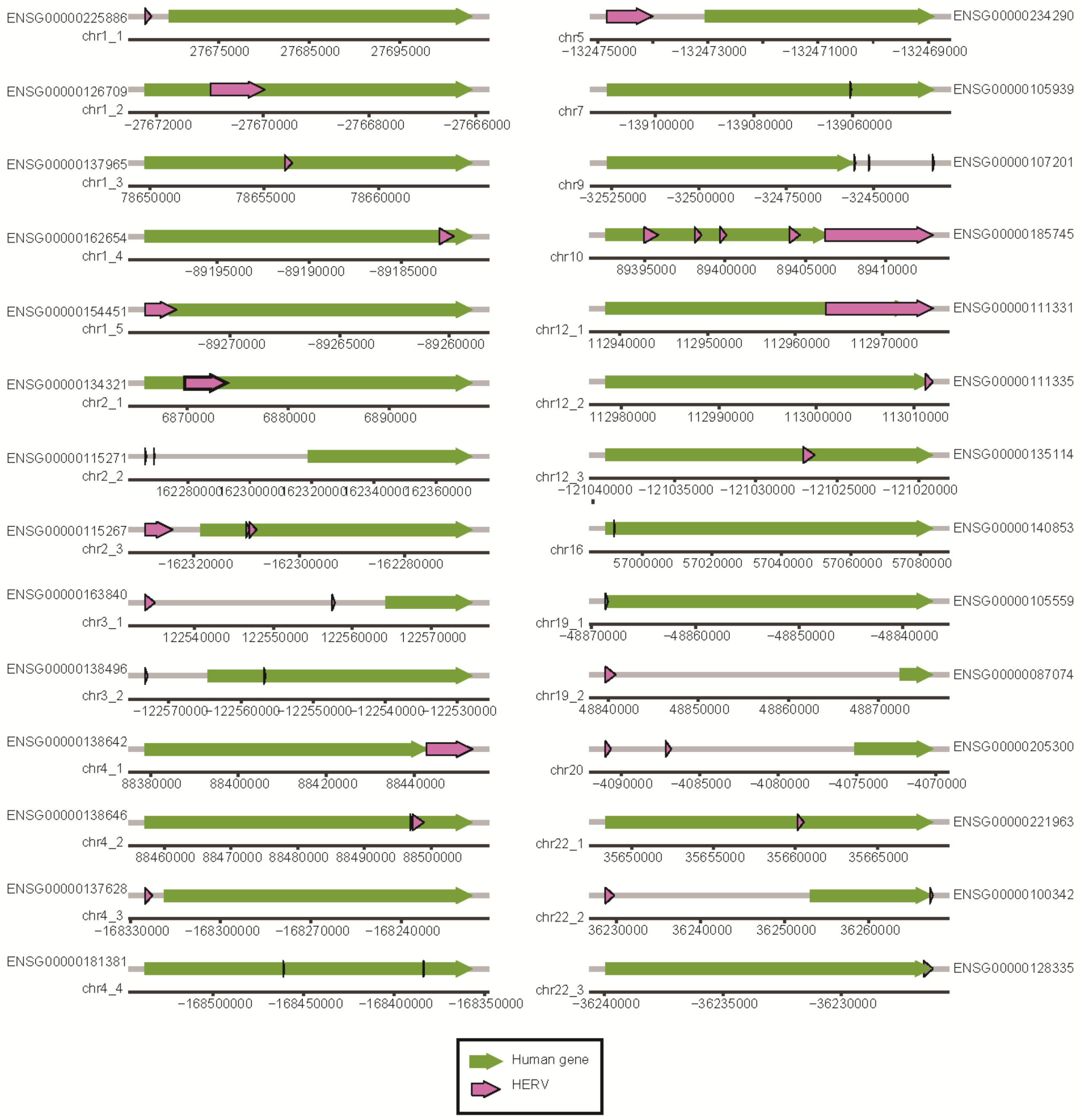

Fig. S3 Distribution of HERVs relative to their paired genes in 43 common pairs.

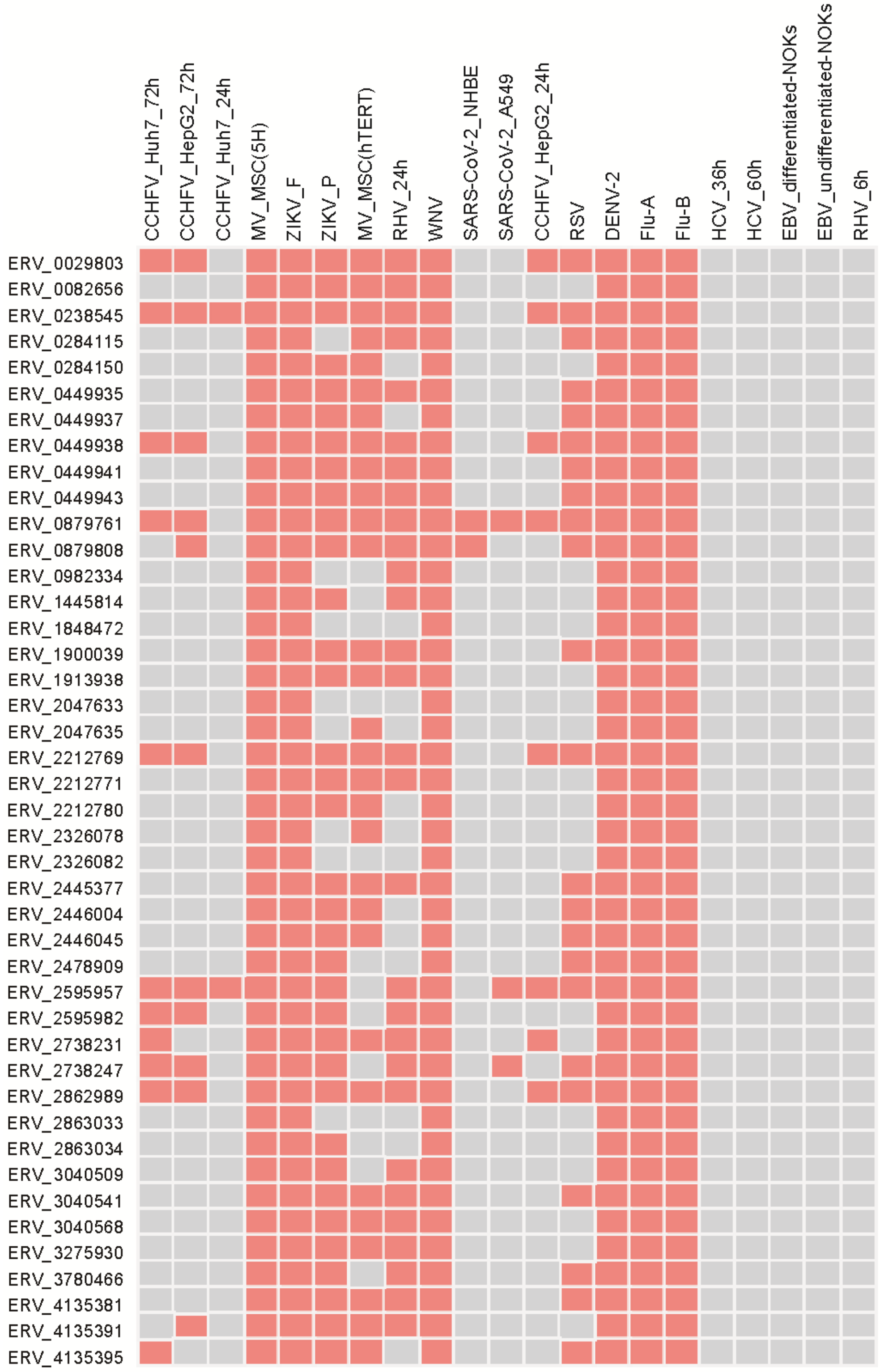

Fig. S4 Distribution of the key DEHERV-G pairs in each dataset.

Table S1. Summary of RNA-seq data from infected and mock cells.

Table S2. Summary of the 43 common DEHERV-G pairs.

Table S3. STRING analysis of the genes of 43 common DEHERV-G pairs.

Table S4. GO and KEGG analysis of the genes of 43 common DEHERV-G
